# Prevalence and early-life risk factors of asthma in preterm adolescents: a cohort study

**DOI:** 10.3389/fped.2025.1720645

**Published:** 2026-01-14

**Authors:** Nienke H. van Dokkum, Arend F. Bos, Sijmen A. Reijneveld, Elianne J. L. E. Vrijlandt

**Affiliations:** 1 Division of Neonatology, Department of Pediatrics, Beatrix Children’s Hospital, University Medical Center Groningen, University of Groningen, Groningen, Netherlands; 2 Department of Health Sciences, University Medical Center Groningen, University of Groningen, Groningen, Netherlands; 3 Division of Pulmonology, Department of Pediatrics, Beatrix Children’s Hospital, University Medical Center Groningen, University of Groningen, Groningen, Netherlands

**Keywords:** adolescence, asthma, epidemiology, prematurity, risk factors

## Abstract

**Aim:**

We seek to elucidate the prevalence, types of respiratory symptoms experienced, and potential early-life risk factors of asthma in adolescence.

**Methods:**

We performed a prospective cohort study including 294 adolescents [130 moderately-late preterm (MLP), 81 early preterm (EP), and 83 full-term (FT)]. Asthma, asthma-like symptoms, and smoking in early childhood and adolescence were self-reported. We collected prenatal and postnatal characteristics, including maternal smoking, bronchopulmonary dysplasia, and respiratory syncytial virus (RSV) infections.

**Results:**

In 11% of EP, 4% of MLP, and 4% of FT adolescents, a formal asthma diagnosis was made. Asthma-like symptoms were reported in 14%, 14%, and 7% of the cases, respectively. Being hospitalized for an RSV infection was associated with a four times higher risk of asthma in adolescence (odds ratio 3.68 and 95% confidence interval 1.04–13.0), while other predictors did not contribute.

**Conclusion:**

MLP adolescents have similar rates of asthma and asthma-like symptoms as their FT peers, while EP adolescents might have a higher risk of asthma but have similar rates of asthma-like symptoms. RSV infections that require hospitalization are associated with an asthma diagnosis in adolescence.

**Clinical Trial Registration:**

https://www.controlled-trials.com, identifier ISRCTN 80622320.

## 1 Introduction

Worldwide, 10% of all births are preterm, i.e., before 37 completed weeks of gestation. Incidence rates vary, estimated as 8%–9% in Europe and 10%–12% in the United States (1). Within the group of preterm births, the largest proportion (approximately 80%–85%) are born moderately-late preterm (MLP), with a gestational age of 32–36 weeks. The remainder are born early preterm (EP), i.e., with a gestational age below 32 weeks (2). While advances in neonatal intensive care treatment have led to a decrease in mortality, short-term and long-term adverse outcomes remain a significant problem (3).

One specific area in which challenges after preterm birth may arise is respiratory health, with preterm birth being a risk factor for the development of respiratory diseases later in life (4). Research shows that children born preterm experience more asthma-like symptoms, including wheezing, coughing, and shortness of breath (5). For EP children, studies have repeatedly shown that asthma-like symptoms are more frequent (6–8). For MLP children, we previously studied respiratory symptoms in a community-based sample during their first 5 years of life and found that they had significantly more asthma-like symptoms than their full-term (FT) peers (9). However, compared with their EP peers, their rates were similar. Others did not report an association between MLP birth and asthma in childhood (10, 11). At later ages, for example, during adolescence, evidence is lacking on asthma and asthma-like symptoms in MLP children. In comparison, for EP children, evidence suggests that asthma and asthma-like symptoms persist (12).

Identification of risk factors can aid in the prediction of asthma and asthma-like symptoms in adolescence. Gestational age may be one of the most important risk factors (13) because at earlier gestational ages, lung structures are more immature and therefore more vulnerable to respiratory diseases. Other pre- and postnatal factors may also increase risk, for example, prenatal exposure to tobacco smoke, receiving ventilator support after birth, having had bronchopulmonary dysplasia (BPD), and respiratory infections, including rhinovirus and respiratory syncytial virus (RSV) (13). With regard to the risk of asthma, the literature commonly reports on RSV, which is associated with recurrent wheezing (14, 15). In addition, the smoking behavior of adolescents may be associated with asthma-like symptoms, especially in preterm adolescents, whose lungs may be vulnerable. In the United States, in the general population, almost one in four high school seniors is a current tobacco smoker, compared with one in three young adults and one in five adults (16).

Understanding the prevalence, nature, and long-term prognosis of respiratory symptoms in preterm adolescents, both early and moderately late, is crucial for finding the best clinical management and improving outcomes. In this study, we seek to elucidate the prevalence and types of respiratory symptoms experienced by these adolescents. Furthermore, we will delve into the potential early life risk factors, such as gestational age and pre- and postnatal factors, along with current exposures that may be associated with asthma in this population.

## 2 Methods

### 2.1 Setting and population

For the current study, we included a subsample of participants from the Longitudinal Preterm Outcome Project (LOLLIPOP), a community-based cohort study of children born between 2002 and 2003, with a main focus on the growth, development, and general health of MLP children compared with those born early preterm and full term. All EP children, MLP children and FT children born in 2002 were included in preventive child health care, while EP children born in 2003 were also included from neonatal intensive care units to enrich the cohort.

For the adolescent follow-up wave of LOLLIPOP, only a part of the original sample was approached, i.e., children born in the three Northern provinces of the Netherlands, because of logistical reasons. This wave was completed between April 2017 and November 2018, when the children were 14–15 years old. In total, 294 adolescents participated, of whom 130 were MLP (response rate 33.0%), 81 were EP (response rate 47.4%), and 83 were FT (response rate 31.1%). There were small differences between responders and non-responders in background characteristics, but with limited effect sizes (17). Parents provided written informed consent for all parts of LOLLIPOP, including follow-up in adolescence. The adolescents themselves also provided written informed consent for participation in the follow-up wave. The LOLLIPOP study and its follow-up waves were approved by the Medical Ethical Review Board of the University Medical Center Groningen (METc 2005/130 and METc 2017/01), the Netherlands.

### 2.2 Respiratory outcomes, potential risk factors, and background

During the adolescent follow-up, parents were asked to complete several questionnaires on health outcomes of their adolescent child, including questions on respiratory health entailing a formal diagnosis of asthma or parent-reported asthma-like symptoms, medication use for asthma, and negative effects of asthma on daily functioning.

Potential risk factors included early life factors and adolescent smoking. With regard to early life factors, we first considered maternal smoking, information was collected through a parental questionnaire completed upon inclusion in the LOLLIPOP cohort. Second, we considered gestational age in weeks, continuous positive airway pressure (CPAP) or mechanical ventilation during the NICU stay, surfactant therapy during the NICU stay, BPD, and RSV infections, with all information extracted from patient records in well-child clinics and NICUs. For RSV, all children who were hospitalized with a proven infection during their first five years of life were classified as having experienced an RSV infection. While others may have also experienced an RSV infection, we hypothesized that it would not be severe enough to affect respiratory outcomes. With regard to the other risk factor, adolescent smoking, information was collected from adolescent reports that were part of a questionnaire on lifestyle that adolescents completed at ages 14–15.

We further collected data on participant characteristics, sex, age at assessment, and maternal educational level. Maternal educational level was dichotomized as low-middle and high, based on the years of education being either 12 years or below or higher than 12 years.

### 2.3 Statistical analysis

First, we assessed differences in participant characteristics using chi-square tests or one-way ANOVA where appropriate. Second, we assessed respiratory outcomes in adolescence in those born MLP, EP, and FT, presenting percentages and testing for differences using chi-square tests for trends. Finally, we performed logistic regression analyses for the risk of asthma in adolescence using the following as predictors in univariable models: maternal smoking during pregnancy, receiving CPAP or mechanical ventilation during the NICU stay, surfactant therapy during the NICU stay, suffering from BPD, experiencing an RSV infection, and smoking. In a multivariable model, we included all statistically significant univariable predictors and adjusted for gestational age category, sex, and being born small for gestational age. All analyses were performed using SPSS version 28.0 (IBM, Armonk, NY, USA). *P*-values <0.05 were considered statistically significant.

## 3 Results

### 3.1 Participant characteristics

A total of 294 adolescents participated in the follow-up wave in adolescence. We present participant characteristics in Table 1. The participating age differed between the participating adolescents, with the EP group being younger than the other two groups. There was no difference in sex or maternal educational level among the groups. Of the 294 participating adolescents and their parents, approximately 20 did not answer the questions on respiratory symptoms in the questionnaires, and some either did not complete the questionnaires at all or left this specific part blank by accidentally skipping this page.

**Table 1:** Participant characteristics in adolescence.

Demographic characteristic	Descriptive	EP	MLP	FT	*p*-value	Non-participants	*p*-value*
		*N* = 81	*N* = 130	*N* = 83		*N* = 537	
Gestational age in weeks	Mean (SD)	29.1 (2.9)	33.9 (1.3)	39.6 (1.9)	<0.001	35.1 (3.8)	0.007
Age at testing in years	Mean (SD)	14.9 (0.8)	15.7 (0.5)	15.5 (0.5)	<0.001	N/A	N/A
Sex	Male subjects, *n* (%)	36 (44.4)	63 (48.5)	37 (44.6)	0.796	299 (55.7)	0.015
	Female subjects, *n* (%)	45 (55.6)	67 (51.5)	46 (55.4)		234 (43.8)	
Maternal education	High,^a^ *n* (%)	24 (29.6)	47 (37.6)	30 (36.6)	0.475	132 (24.6)	0.005
	Low–middle, *n* (%)	57 (70.4)	78 (62.4)	52 (63.4)		387 (72.1)	

N/A, not applicable.

a High maternal education was defined as an educational level of>12 years.

* *p*-value comparing participating adolescents vs. non-participating adolescents.

### 3.2 Respiratory symptoms

We present respiratory outcomes in adolescence in Table 2. The proportion of parents reporting a formal diagnosis of asthma was 11% of EP adolescents vs. 4% in both MLP and FT adolescents (*p* = 0.064). Of adolescents with a parent-reported asthma diagnosis or asthma-like symptoms (*n* = 32, of whom 10 were born EP, 17 MLP, and 5 FT), the majority reported using asthma medication in the 12 months before completing the questionnaire, and up to 40% reported negative effects of asthma on daily life. In contrast to the findings regarding parent-reported formal diagnoses, a higher proportion of preterm-born adolescents experienced wheezing symptoms up to and during adolescence. Of all adolescents who ever experienced wheezing, fewer than 5 adolescents per gestational age group experienced wheezing during the year before the questionnaire was completed.

**Table 2:** Respiratory outcomes in adolescence.

Respiratory outcome	EP	MLP	FT	*p*-value*
Parent-reported formal diagnosis of asthma up to and including adolescence (*n* = 276)	8/73 (11%)	5/124 (4%)	3/79 (4%)	0.064
Parent-reported asthma or asthma-like symptoms up to and including adolescence (*n* = 266)	10/69 (14%)	17/120 (14%)	5/77 (7%)	0.196
Asthma medication in the 12 months before the study visit (*n* = 31)	6/10 (60%)	5/16 (31%)	3/5 (60%)	0.697
Negative effects of asthma on daily functioning (*n* = 28)	4/10 (40%)	1/11 (9%)	2/5 (40%)	0.728
Ever experienced wheezing symptoms (*n* = 273)	18/70 (26%)	23/123 (19%)	7/80 (9%)	0.006
Experienced wheezing in the year before the study (*n* = 46)	2/18 (11%)	4/21 (19%)	3/7 (43%)	0.097

* *p*-value of chi-square test for trends.

### 3.3 Risk of asthma development at adolescence

**Figure 1:**
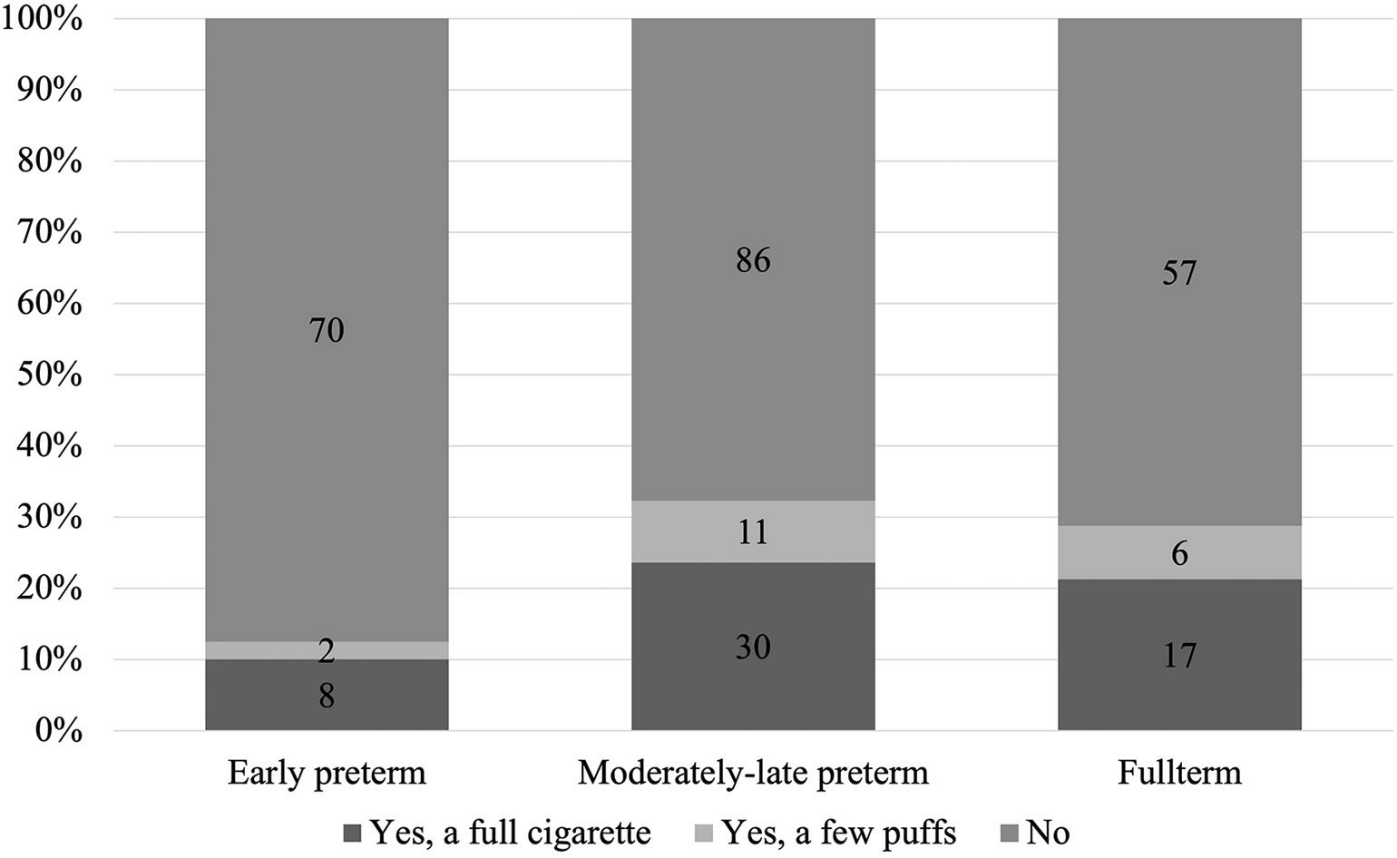
Adolescent responses to being asked “have you ever smoked a cigarette” by gestational age category (chi-square test for trends *p* = 0.029).

It was found that in total, 14% of mothers smoked during pregnancy. During the NICU stay, 30% of adolescents received CPAP or mechanical ventilation, and 12% received surfactants, and the majority of them were born EP. Of the EP children, 8% developed BPD. Of all adolescents, 8% was hospitalized for an RSV infection. With regard to smoking, fewer EP adolescents reported having ever smoked, compared to their MLP and FT peers (Figure 1). Current smoking rates were much lower and did not differ statistically between gestational age categories (chi-square test for trends, *p* = 0.458).

The results of logistic regression analyses on the risk of asthma in adolescence about the aforementioned factors, are presented in Table 3. In univariable analyses, for those who had experienced an RSV infection that required hospitalization, the risk of asthma in adolescence was four times higher, and for those who had suffered from BPD, it was two times higher. After adjustments for gestational age category, sex, and being born small for gestational age, the risk of asthma in adolescence remained four times higher for those with an RSV infection requiring hospitalization. For those with a history of BPD, the magnitude remained similar but failed to reach statistical significance.

**Table 3:** Logistic regression analysis for risk of asthma in adolescence in EP children.

Risk factor	Univariable model		Multivariable model*	
	OR (95% CI)	*p*-value	OR (95% CI)	*p*-value
Maternal smoking during pregnancy	0.43 (0.09–1.95)	0.27	–	–
CPAP or mechanical ventilation during NICU stay	2.21 (0.81–6.07)	0.12	–	–
Surfactants during NICU stay	2.03 (0.66–6.31)	0.22	–	–
Suffering from BPD	1.98 (0.98–4.03)	0.059	2.17 (0.88–5.34)	0.094
Experienced RSV infection before age 5	4.06 (1.29–12.8)	0.017	3.68 (1.04–13.0)	0.043
Having ever smoked	1.40 (0.63–3.14)	0.409	–	–

CPAP, continuous positive airway pressure; NICU, neonatal intensive care unit; BPD, bronchopulmonary dysplasia; RSV, respiratory syncytial virus.

* Multivariable analysis considered a model with a univariable predictor (if statistically significantly related to the risk of asthma in adolescence, *p* < 0.10), adjusted for sex, gestational age, and being born small for gestational age.

## 4 Discussion

In this study, we aimed to understand the prevalence of respiratory symptoms in preterm adolescents, with a special interest in MLP adolescents, and to identify potential risk factors for asthma. This study demonstrated that MLP children experienced more wheezing symptoms during their life but had similar rates of asthma and asthma-like symptoms as their FT counterparts. EP adolescents had the highest prevalence of wheezing symptoms and had a higher prevalence of asthma compared with their FT counterparts. We found RSV infections that require hospitalization to be an independent risk factor for asthma in adolescence, while other predictors did not emerge.

We found a similar prevalence of respiratory symptoms between MLP adolescents and their FT peers, but a higher prevalence among EP adolescents. This is in line with the results of a Swedish study, which also reported an approximately twofold higher risk of asthma in preterm young adults between 25 and 35 years of age who were born between 23 and 27 weeks of gestation, but not in preterm children born after a longer gestation period (18). Our findings were also recently confirmed by a Spanish population–based study that included 13–14-year-old adolescents (19). Possible mechanisms underlying the increased prevalence of asthma and asthma-like symptoms in children and adolescents born preterm include altered lung growth and associated structural changes (20). In preterm children, altered lung growth can lead to a reduction in peak lung function (4) and subsequent vulnerability to external exposures. Postnatal exposures, such as respiratory support, may cause inflammation in the lung that can alter the structure of the airways, also known as remodeling. In case of birth at lower gestational ages, lung growth *in utero* is interrupted earlier, and more respiratory support is needed, making the youngest preterm children more impacted (21) and thereby explaining the differences in asthma and asthma-like symptoms between EP and MLP children.

We found RSV infections that require hospitalization to be an independent risk factor for asthma in adolescence. To the best of our knowledge, ours is the first study to report on this association regarding outcomes in adolescence. Our findings align with the available evidence on infancy and childhood, as reviewed by Szabo et al. (22), and in more recent studies by Ruotsalainen et al. (23) and Rosas-Salazar et al. (24). The proposed immunological mechanism involves antiviral interferon-gamma-mediated immunity that is shifted toward a T-helper-2 cell-dominant response, resulting in a more asthma-prone airway, with hyperplasia and hyperreactivity, in preterm infants (25). Genetic and environmental factors are also hypothesized to be involved (25). This association between RSV infections and asthma may call for the prevention of RSV infections through immunization.

We found BPD to be a predictor in our univariable model but not in our multivariable model. With regard to BPD as a predictor, other studies have identified an independent relationship with asthma, especially studies focusing on EP adolescents (19, 26), but we also identified one study confirming our results (27). Interestingly, BPD appears to be mainly predictive for lung function, i.e., forced expiratory volume in 1 s (FEV1), rather than asthmatic symptoms (28). However, as impaired lung function is one of the mechanisms underlying airway remodeling, it is plausible that BPD is a predictor of asthma and asthma-like symptoms. An explanation for our somewhat ambiguous findings may be that the sample size of our study was too small to consistently identify an association with BPD.

While examining current smoking behavior as a predictor of asthma, we surprisingly observed that MLP adolescents smoked more often than their EP peers. Evidence on the risk-taking behavior of preterm-born adolescents, including smoking, is limited. The few available studies show low smoking rates among EP adolescents compared to the general population (29) or no differences at all (30). These lower rates may be explained by the increased parental monitoring and the influence of the family environment that arises from preterm birth (31). We did not identify studies on MLP adolescents and their risk-taking behaviors. Future studies should assess risk-taking behaviors in this specific group, and when the findings are confirmed, paying attention to such behaviors early in follow-up care may be indicated, as the representation of MLP-born individuals in society is significant.

We were also surprised that we did not identify smoking, either maternal or current, as a predictor for asthma or asthma-like symptoms in adolescence. This serves as a contrast to current evidence, which shows that prenatal and passive smoke exposures increase the risk of asthma and asthma-like symptoms, particularly in early childhood (32). This failure of identification may be due to the limited statistical power of our study, as the suggested mechanisms are quite plausible. These suggested mechanisms consider the fact that prenatal smoking leads to altered lung function, with direct effects on alveolar cells, fibroblasts, endothelial cells, and stem cells, primarily through exposure to nicotine (32, 33). Additionally, prenatal smoking may alter DNA methylation in the placenta and fetus in specific genes, leading to increased susceptibility to the development of asthma (33).

### 4.1 Strengths and limitations

The main strength of this study is its inclusion of MLP children, for whom follow-up data during adolescence and young adulthood are limited. However, we also acknowledge several limitations in this study. First, this study may have insufficient statistical power to detect associations between risk factors that were relatively uncommon in our cohort and asthma or asthma-like symptoms in adolescence. Data on risk factors were also available for a variable number of adolescents. Combined with a fairly low response rate to our questionnaires at this point in the follow-up (30%–40%), which may have introduced selection bias, our findings should be interpreted with caution. Regarding RSV infection, we did not have data on whether all children were tested during hospitalization, which may have led to an underestimation of its effects. Second, we relied on self-reports of symptoms and diagnoses, and also of risk factors, and did not perform lung function tests in adolescence. However, based on literature findings, we know that those born MLP have only slightly reduced lung function compared with their FT peers (34).

### 4.2 Implications

Our findings imply that MLP adolescents do not suffer from asthma or asthma-like symptoms more often than the general population. However, for those born EP, clinical follow-up care should include asthma and asthma-like symptoms, as they do have a higher prevalence of asthma even in adolescence. Additionally, for those children with symptoms in early childhood, follow-up care may be continued into adolescence, as these are highly predictive of sustained symptoms. Furthermore, smoking, either prenatally or currently, should be avoided as much as possible, as this may lead to an increased susceptibility to asthma and asthma-like symptoms. Additional research should confirm the findings of this study in larger cohorts, especially with regard to risk factors for asthma in adolescence.

## 5 Conclusion

MLP children experience more wheezing symptoms during their life, but in adolescence, they have similar rates of asthma and asthma-like symptoms as their FT peers, while EP adolescents have a higher risk of asthma or asthma-like symptoms. In addition, RSV infections that require hospitalization are independently associated with asthma in adolescence, while other predictors do not emerge.

## Data Availability

The raw data supporting the conclusions of this article will be made available by the authors without undue reservation.
